# Kaposi’s Sarcoma-Associated Herpesvirus ORF50 Protein Represses Cellular MDM2 Expression via Suppressing the Sp1- and p53-Mediated Transactivation

**DOI:** 10.3390/ijms23158673

**Published:** 2022-08-04

**Authors:** Chia-I Lin, Shie-Shan Wang, Chien-Hui Hung, Pey-Jium Chang, Lee-Wen Chen

**Affiliations:** 1 Graduate Institute of Clinical Medical Sciences, College of Medicine, Chang-Gung University, Taoyuan 33302, Taiwan; 2 Department of Pediatric Surgery, Chang-Gung Memorial Hospital, Chiayi 61363, Taiwan; 3 School of Medicine, Chang-Gung University, Taoyuan 33302, Taiwan; 4 Department of Nephrology, Chang-Gung Memorial Hospital, Chiayi 61363, Taiwan; 5 Department of Respiratory Care, Chang-Gung University of Science and Technology, Chiayi 61363, Taiwan

**Keywords:** KSHV, ORF50, MDM2, Sp1, p53, transcriptional repression

## Abstract

The Kaposi’s sarcoma-associated herpesvirus (KSHV)-encoded ORF50 protein is a potent transcriptional activator essential for triggering KSHV lytic reactivation. Despite extensive studies, little is known about whether ORF50 possesses the ability to repress gene expression or has an antagonistic action to cellular transcription factors. Previously, we demonstrated that human oncoprotein MDM2 can promote the degradation of ORF50 protein. Herein, we show that abundant ORF50 expression in cells can conversely downregulate MDM2 expression via repressing both the upstream (P1) and internal (P2) promoters of the MDM2 gene. Deletion analysis of the MDM2 P1 promoter revealed that there were two ORF50-dependent negative response elements located from −102 to −63 and from −39 to +1, which contain Sp1-binding sites. For the MDM2 P2 promoter, the ORF50-dependent negative response element was identified in the region from −110 to −25, which is coincident with the location of two known p53-binding sites. Importantly, we further demonstrated that overexpression of Sp1 or p53 in cells indeed upregulated MDM2 expression; however, coexpression with ORF50 protein remarkably reduced the Sp1- or p53-mediated MDM2 upregulation. Collectively, our findings propose a reciprocal negative regulation between ORF50 and MDM2 and uncover that ORF50 decreases MDM2 expression through repressing Sp1- and p53-mediated transactivation.

## 1. Introduction

Kaposi’s sarcoma-associated herpesvirus (KSHV), also known as human herpesvirus 8 (HHV8), is implicated as the causative agent of several human malignancies, including Kaposi’s sarcoma (KS), primary effusion lymphoma (PEL), and multicentric Castleman’s disease [1,2,3]. Like other herpesviruses, KSHV exhibits two distinct life cycles, latency and the lytic cycle [4,5]. The switch of KSHV from latency to lytic replication is initiated by the expression of a viral regulator encoded from the open reading frame 50 (ORF50) [6,7]. The ORF50 protein, also known as RTA (replication and transcription activator), is a 691-amino-acid (aa) transactivator with an N-terminal basic DNA-binding domain (aa 1 to 390) and a C-terminal acidic activation domain (aa 486 to 691) [8,9]. Numerous studies have shown that ORF50 protein can use different strategies to transcriptionally activate a considerable number of viral and cellular gene promoters [10,11,12,13,14]. In addition to acting as a transcriptional activator, ORF50 protein can also functions as a replication factor critically involved in the recruitment of the prereplication complex to the lytic origin of replication (oriLyt) [13,15]. Furthermore, ORF50 protein has been previously reported as an ubiquitin E3 ligase that may promote the degradation of its interacting target proteins via the ubiquitin–proteasome system [16,17]. Since ORF50 is a multifunctional protein and plays a key role in the latent-to-lytic switch, it is possible that ORF50 may have more functions than we expected.

Murine double-minute 2 homolog (MDM2) is an oncoprotein with the function of ubiquitin E3 ligase [18]. Upregulation of MDM2 has been reported in a variety of human cancers, which critically correlated with cancer progression and chemotherapeutic resistance [19]. MDM2 is known to bind and ubiquitinate the p53 tumor suppressor, which results in inactivation and degradation of the p53 protein [20,21]. Although MDM2 is a negative regulator of p53, the expression of MDM2 can be transcriptionally activated by p53 [20,22]. Transcription of the MDM2 gene is controlled by two distinct promoters, namely P1 and P2 [20,22,23]. The P1 promoter of the MDM2 gene located upstream of exon 1 is mainly responsible for the basal constitutive MDM2 expression, whereas the P2 promoter located in the first intron is involved in a stress-inducible MDM2 expression. Particularly, the P2 promoter contains two p53-binding sites (or p53-responsive elements, p53-REs) that confer p53 responsiveness in various pathophysiological conditions [20,22].

In addition to having pro-oncogenic functions in cells, increasing evidence has implicated that MDM2 may play a critical role in regulating the replication of different DNA and RNA viruses including KSHV [24,25], adenovirus [26], human immunodeficiency virus [27,28], influenza A virus [29], and Zika virus [30]. For KSHV, several studies have suggested that MDM2 acts as a negative regulator of the viral lytic reactivation. Previously, Ye et al. [31] noticed that treatment of a xenograft mouse model of Kaposi’s sarcoma with Nutlin-3, an MDM2 inhibitor, significantly induced the expression of the viral lytic genes. Balistreri et al. [24] also reported that the depletion of MDM2 by siRNAs in a KSHV-positive cell line iSLK.219 facilitated efficient induction of lytic reactivation. Consistent with this notion, our earlier studies demonstrated that MDM2 is capable of interacting with ORF50 protein and promoting ORF50 degradation through the ubiquitin–proteasome pathway [25]. Thus, in latently KSHV-infected cells, abundant MDM2 expression was suggested to restrict the basal protein level of ORF50. In our previous studies, we additionally found that when PEL cells were induced to enter the KSHV lytic cycle by chemical agents, the protein levels of MDM2 could be dramatically diminished in the lytic cycle [25]. Currently, the causal relationship between KSHV lytic reactivation and MDM2 downregulation in cells after treatment with chemical-inducing agents still needs to be further clarified. Additionally, it remains largely unclear whether specific viral factors could be involved in the downregulation of MDM2 in the viral lytic cycle.

In this report, we demonstrated that KSHV lytic reactivation could lead to MDM2 downregulation and identified that ORF50 protein was a key factor in repressing MDM2 expression at the transcription level. Mechanistic studies revealed that ORF50 protein suppressed both the P1 and P2 promoters of the MDM2 gene through affecting Sp1- and p53-mediated transactivation, respectively. Overall, our findings propose a negative regulatory relationship between ORF50 and MDM2 expression and show that ORF50 protein has an antagonistic action to specific cellular transcription factors.

## 2. Results

### 2.1. MDM2 Is Downregulated during the KSHV Lytic Cycle

**Figure 1:**
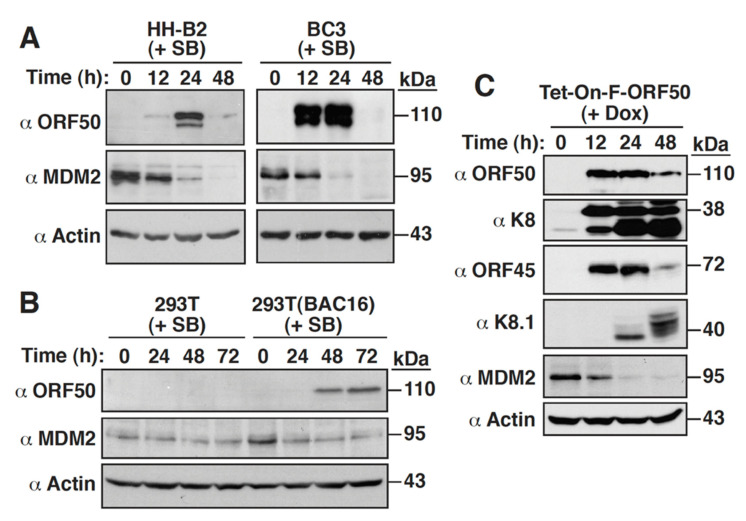
Downregulation of MDM2 occurs during the KSHV lytic cycle. (**A**) Expression kinetics of MDM2 in HH-B2 and BC3 cells during the KSHV lytic cycle. The expression of MDM2 and the viral lytic protein ORF50 was analyzed by Western blotting in HH-B2 and BC3 cells after treatment with sodium butyrate (SB) for 12, 24, and 48 h. (**B**) Western blot analysis of MDM2 expression in 293T and 293T(BAC16) cells after treatment with SB. At the indicated time points after SB treatment, the expressions of MDM2 and ORF50 in the treated cells were determined by immunoblotting. (**C**) Western blot analysis of MDM2 and viral lytic proteins expressed in HH-B2(Tet-On-F-ORF50) cells after treatment with doxycycline (Dox). Note that the viral proteins ORF50, K8, and ORF45 belong to the immediate-early or early lytic proteins, whereas K8.1 is a late lytic protein.

Previously, we showed that treatment of PEL cell lines including HH-B2 and BC3 with sodium butyrate (SB), a KSHV lytic cycle-inducing agent, remarkably downregulated MDM2 expression [25]. As shown in Figure 1A, we confirm that in both HH-B2 and BC3 cells, SB treatment could activate the expression of the viral immediate-early protein ORF50 and downregulate MDM2 expression at early time points. To further investigate the association between KSHV lytic reactivation and MDM2 downregulation, we evaluated the effects of SB on MDM2 expression in KSHV-negative 293T cells and 293T(BAC16) cells that carry the recombinant KSHV genome BAC16. Western blot analysis revealed that no significant changes in MDM2 levels were observed in 293T cells following SB treatment; however, MDM2 expression was significantly diminished in 293T(BAC16) cells after SB treatment (Figure 1B). Due to the concomitant expression of ORF50 in SB-treated 293T(BAC16) cells (Figure 1B), our results strongly suggest that downregulation of MDM2 by SB was associated with KSHV lytic reactivation. Since chemical-inducing agents, such as SB, could probably cause profound effects on cellular gene expression, a stable HH-B2 cell clone Tet-On-F-ORF50 that contains an exogenously doxycycline-regulated ORF50 gene was used in the study. Our results show that treatment of HH-B2(Tet-On-F-ORF50) cells with doxycycline resulted in the induction of ORF50 protein and its downstream viral proteins including K8, ORF45, and K8.1, which was also accompanied by the downregulation of MDM2 (Figure 1C). Collectively, our findings demonstrate that KSHV lytic reactivation could lead to MDM2 downregulation in PEL cells.

### 2.2. ORF50 Protein Downregulates MDM2 Expression Independently of Other Viral Factors

**Figure 2:**
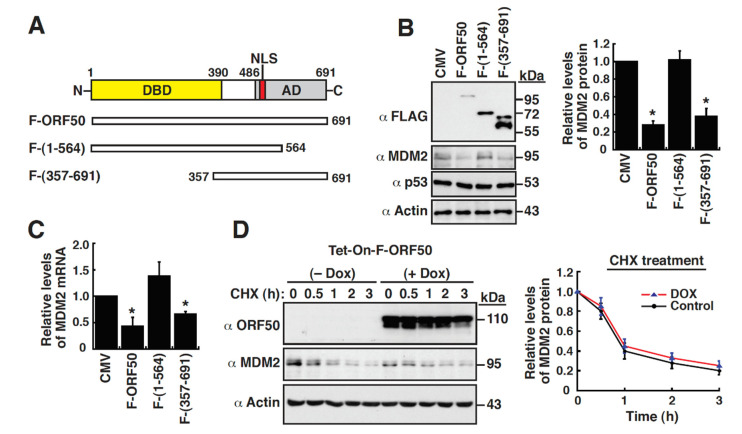
ORF50 transcriptionally downregulates MDM2 expression independently of other viral proteins. (**A**) Schematic diagram of ORF50 and its deletion constructs. Specific functional domains or motifs of ORF50 protein are indicated as follows: DBD, DNA-binding domain (aa 1–390); AD, activation domain, (aa 486–691); NLS, nuclear localization signal. (**B**) Effect of wild-type or mutant ORF50 on the expression of MDM2 and p53 in 293T cells. 293T cells were transfected with the empty vector or different ORF50 constructs, and then the expression of the indicated proteins in transfected cells was examined by Western blotting. Quantitative data of MDM2 expression in transfected cells are indicated as mean ± SEM (n = 3). *, *p* < 0.05, for results compared to those with the empty vector control. (**C**) Quantitative RT-PCR analysis of the MDM2 mRNA levels in 293T cells transfected with the empty vector or different ORF50 constructs. Levels of the MDM2 mRNAs in transfected cells were normalized with the levels of 18S rRNA. *, *p* < 0.05, for results compared to those with the empty vector control (n = 3). (**D**) Analysis of protein stability of MDM2 in HH-B2(Tet-On-F-ORF50) cells by cycloheximide chase assays. HH-B2(Tet-On-F-ORF50) cells were left untreated or treated with doxycycline for 16 h, and then the cells were treated with cycloheximide for another 0.5, 1, 2, and 3 h. Western blots for MDM2 expression were quantified by densitometry (n = 3; right panel).

We next determined whether ORF50 alone could repress MDM2 expression independently of other viral factors. Accordingly, 293T cells were transfected with the empty vector (CMV) or the expression plasmid encoding FLAG-tagged ORF50 (F-ORF50) or its deletion mutants (Figure 2A). Western blot analysis showed that F-ORF50 expression in 293T cells was able to downregulate MDM2 expression (Figure 2A,B); however, the expression of the ORF50 DNA-binding domain from aa 1 to 564, F-ORF50(1-564), in 293T cells failed to reduce MDM2 expression (Figure 2A,B). On the other hand, overexpression of F-ORF50 or F-ORF50(1-564) in 293T cells did not affect the expression levels of p53 in the same conditions (Figure 2B). These results indicate that ORF50 specifically repressed MDM2 expression, without the need of other viral proteins. Noteworthily, the ORF50 C-terminal activation domain from aa 357 to 691, F-ORF50(357-691), could also reduce the protein level of MDM2 in 293T cells, albeit less efficiently than the full-length F-ORF50 (Figure 2A,B). When mRNA expression levels of MDM2 in transfected cells were analyzed by quantitative reverse transcription polymerase chain reaction (RT-qPCR), we consistently found that the MDM2 mRNA levels were reduced by 66% and 40% in the F-ORF50- and F-ORF50(357-691)-transfected cells, respectively, as compared to the empty vector-transfected cells (Figure 2C). In contrast, overexpression of F-ORF50(1-564) in 293T cells did not cause the reduction of MDM2 mRNA levels (Figure 2C). Since ORF50 was previously reported as an ubiquitin E3 ligase and could interact with MDM2, it was possible that ORF50 might also decrease MDM2 levels by promoting its protein degradation. To clarify this point, we performed cycloheximide chase assays to monitor the protein levels of MDM2 in the presence or absence of ORF50. As shown in Figure 2D, ORF50 expression in HH-B2(Tet-On-F-ORF50) cells did not display an increased MDM2 degradation. These results suggest that downregulation of MDM2 expression by ORF50 was mainly mediated at the transcriptional level.

### 2.3. Both the Upstream Promoter (P1) and the Internal Promoter (P2) of the MDM2 Gene Are Repressed by ORF50

**Figure 3:**
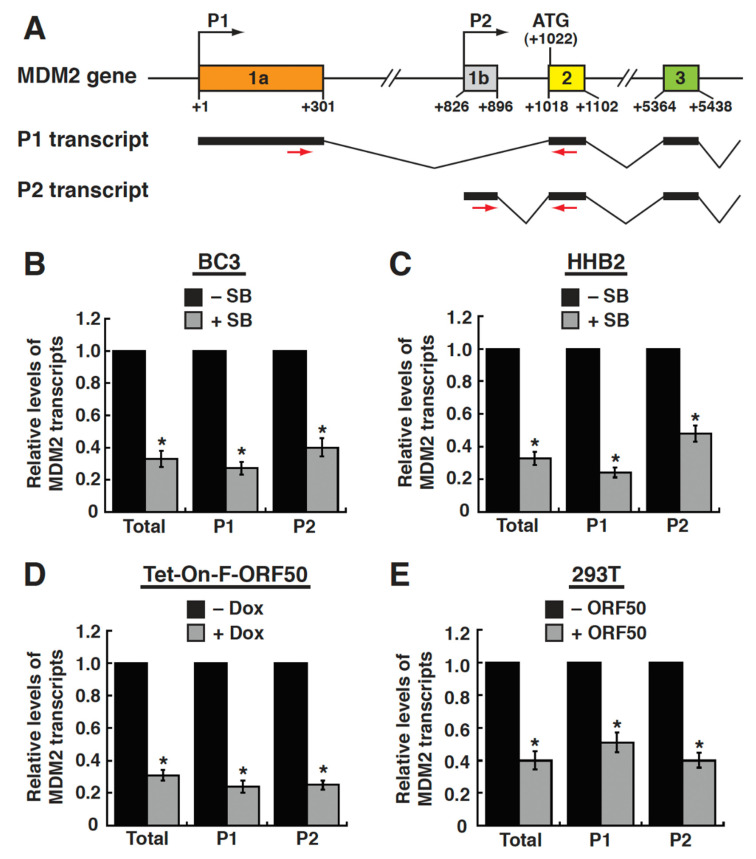
ORF50 represses both the P1 and P2 promoters of the MDM2 gene. (**A**) Schematic diagram of the P1 and P2 promoter regions of the MDM2 gene. The color boxes in the diagram indicate exons, and the nucleotide positions are numbered with respect to the transcription start site of the P1 promoter. The P1- and P2-derived mRNA transcripts are also included in the diagram. Small red arrows depict the positions of the primers for quantitative RT-PCR. (**B**) Relative levels of total MDM2 mRNAs and the P1- and P2-derived MDM2 mRNAs in BC3 cells untreated or treated with SB for 24 h. *, *p* < 0.05, for results compared to those from the untreated group (n = 3). (**C**) Relative levels of total MDM2 mRNAs and the P1- and P2-derived MDM2 mRNAs in HH-B2 cells untreated or treated with SB for 24 h. *, *p* < 0.05, for results compared to those from the untreated group (n = 3). (**D**) Relative levels of total MDM2 mRNAs and the P1- and P2-derived MDM2 mRNAs in HH-B2(Tet-On-F-ORF50) cells untreated or treated with doxycycline for 24 h. *, *p* < 0.05, for results compared to those from the untreated group (n = 3). (**E**) Relative levels of total MDM2 mRNAs and the P1- and P2-derived MDM2 mRNAs in 293T cells transfected with the empty vector or the ORF50 expression plasmid for 24 h. *, *p* < 0.05, for results compared to those from the empty vector group (n = 3).

Previous studies have shown that the transcription of the MDM2 gene occurs at two distinct promoters, namely P1 and P2 [20,22]. To determine which promoter of the MDM2 gene was responsive to ORF50, both the P1-and P2-derived mRNA transcripts were measured by RT-qPCR in different cell lines (Figure 3A). When SB was added to induce ORF50 expression in BC3 and HH-B2 cells, we found that the levels of both the P1- and P2-derived mRNAs were significantly reduced in these SB-treated cells compared to untreated cells (Figure 3B,C). Moreover, the induction of ORF50 expression in HH-B2(Tet-On-F-ORF50) cells by doxycycline also revealed lower levels of mRNA transcripts from both the P1 and P2 promoters (Figure 3D). Importantly, we also found that the levels of both the P1- and P2-derived mRNA transcripts were significantly reduced in ORF50-transfected 293T cells, in comparison with the empty vector-transfected cells (Figure 3E). These results suggest that ORF50 protein could negatively regulate both the P1 and P2 promoters of the MDM2 gene.

**Figure 4:**
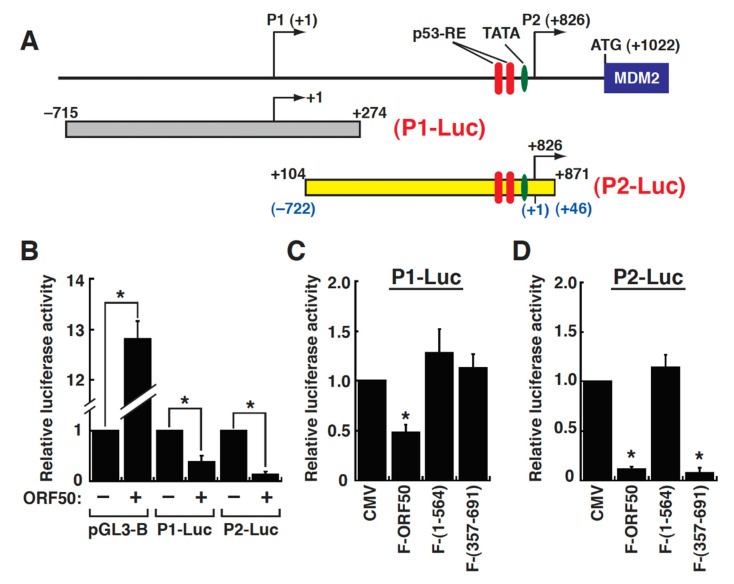
Analysis of the regulation of both the MDM2 P1 and P2 promoters using luciferase reporter gene assays. (**A**) Schematic diagram of the reporter constructs with the P1 or P2 promoter of the MDM2 gene. The P1 promoter from –715 to +274 and the P2 promoter from –722 to +46 (or +104 to +871 with respect to the P1 transcription start site) were individually cloned into pGL3-Basic, resulting in the generation of P1-Luc and P2-Luc, respectively. Note that there are two p53-responsive elements (p53-RE) in the P2 promoter region. (**B**) Effect of ORF50 on the reporter gene expression of pGL3-Basic, P1-Luc, and P2-Luc in 293T cells. After 293T cells were cotransfected with the indicated reporter constructs and the ORF50-expressing plasmid or the empty vector control (CMV) for 30 h, cells were harvested and assayed for luciferase activity. *, *p* < 0.05, for results compared to those from the empty vector group (n = 3). (**C**) Repression of the MDM2 P1 promoter by ORF50 and its deletion mutants. The P1-Luc reporter construct was cotransfected with the indicated expression plasmids into 293T cells. At 30 h after transfection, cells were harvested, and luciferase activity was measured. *, *p* < 0.05, for results compared to those from the empty vector group. (**D**) Repression of the MDM2 P2 promoter by ORF50 and its deletion mutants. *, *p* < 0.05, for results compared to those from the empty vector group.

To further confirm this notion, the P1 promoter region from –715 to +274 and the P2 promoter region from −722 to +46 (or +104 to +871 with respect to the P1 transcription start site) were individually cloned into a luciferase reporter plasmid pGL3-Basic, resulting in the generation of P1-Luc and P2-Luc, respectively (Figure 4A). The responses of the P1-Luc and P2-Luc constructs to ORF50 in 293T cells were then evaluated by transient reporter assays. Normally, we found that the control plasmid pGL3-Basic had a high background reporter activation in response to ORF50 (up to 13-fold) in 293T cells (Figure 4B). When the P1-Luc and P2-Luc reporter constructs were cotransfected with the ORF50 expression plasmid in 293T cells, the reporter expression of both reporter constructs was conversely suppressed by ORF50 (Figure 4B). It was worth noting that for the P1-Luc reporter construct, neither F-ORF50(1-564) nor F-ORF50(357-691) inhibited the reporter gene expression in 293T cells (Figure 4C). Interestingly, although F-ORF50(357-691) failed to inhibit the reporter expression of the P1-Luc reporter construct, this ORF50 mutant still retained the ability to repress the reporter expression of the P2-Luc reporter construct (Figure 4D). These results imply that ORF50 protein might repress the P1 and P2 promoters of the MDM2 gene through different mechanisms.

### 2.4. Mapping of the ORF50-Dependent Negative Response Elements in the P1 Promoter of the MDM2 Gene

**Figure 5:**
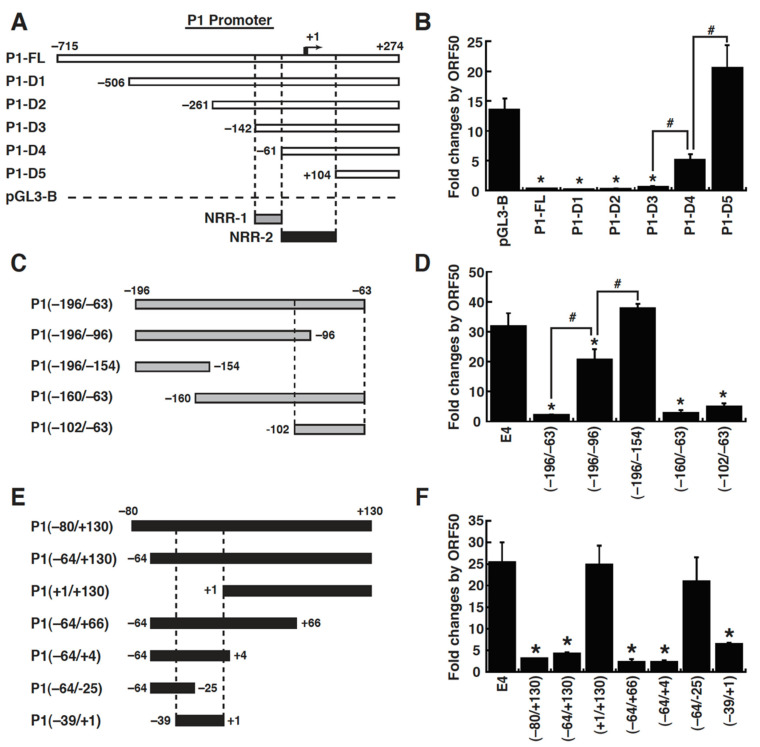
Mapping of the ORF50-dependent negative response element in the P1 promoter of the MDM2 gene. (**A**) Schematic diagram of the promoter–reporter constructs with sequential deletions of the P1(–715/+274) promoter region. Two promoter regions, including NRR-1 and NRR-2, critical for ORF50-mediated repression are shown in the diagram. (**B**) Responsiveness of the reporter constructs with the P1 promoter regions to ORF50 in 293T cells. The indicated reporter constructs were cotransfected with the empty vector or the ORF50-expressing plasmid into 293T cells. After 30 h, cells were lysed, and the luciferase reporter activity was measured. Fold change data of reporter constructs were calculated as the luciferase activity in the presence of ORF50 divided by the luciferase activity in the absence of ORF50. *, *p* < 0.05, compared to pGL3-Basic; #, *p* < 0.05, compared to the indicated reporter constructs (n = 3). (**C**,**D**) Fine mapping of a minimal ORF50-dependent negative response element in the P1(–196/–63) region. The P1(–196/–63) promoter region and its 5′ or 3′ deletions in the diagram were cloned into pE4-luc. The responsiveness of the indicated reporter constructs to ORF50 was determined in 293T cells. Fold change data of these reporter constructs were calculated as the luciferase activity in the presence of ORF50 divided by the luciferase activity in the absence of ORF50. *, *p* < 0.05, compared to pGL3-Basic; #, *p* < 0.05, compared to the indicated reporter constructs (n = 3). (**E**,**F**) Fine mapping of a minimal ORF50-dependent negative response element in the P1(–80/+130) region. The P1(–80/+130) promoter region and its deletion fragments shown in the diagram were cloned into pE4-luc. The responsiveness of the indicated reporter constructs to ORF50 was analyzed in 293T cells. *, *p* < 0.05, compared to pE4-luc (n = 3).

To elucidate the mechanism of action of ORF50 in repressing the MDM2 P1 promoter, we first mapped the ORF50-dependent negative response element in the P1 promoter. To this end, a series of 5′ deletions of the P1 promoter were constructed into pGL3-Basic, and their responses to ORF50 were evaluated in 293T cells. Notably, the MDM2 P1 promoter is a TATA-less promoter that contains GC-rich sequences in its core promoter region. Deletion analysis showed that there were two ORF50-dependent negative response regions in the P1 promoter critical for the ORF50-mediated repression, which were located from –142 to –61 (designated as NRR-1, negative response region 1) and from –61 to +104 (designated as NRR-2, negative response region 2) (Figure 5A,B). To further verify the identified *cis*-acting regions in the P1 promoter, the promoter regions from –196 to –63 and from –80 to +130, which cover the NRR-1 and NRR-2 regions, respectively, were individually cloned into pE4-luc, a reporter construct with a minimal E4 promoter of adenovirus. The resultant reporter plasmids, P1(–196/–63)/E4 and P1(–80/+130)/E4, were then cotransfected with the ORF50 expression plasmid or the empty vector in 293T cells, and the luciferase reporter assays were performed. Compared to the control pE4-luc, we found that both the P1(–196/–63)/E4 and P1(–80/+130)/E4 reporter constructs were significantly repressed by ORF50 (Figure 5C–F). To further map a minimal element required for responding to ORF50 in the P1(–196/–63) and P1(–80/+130) promoter regions, deletion mutants were made from both the 5′ and 3′ ends of these two promoter regions. Transient reporter analysis revealed that the P1(–102/–63) motif located in the NRR-1 region and the P1(–39/+1) motif located in the NRR-2 region were identified as the ORF50-dependent negative response elements (Figure 5C–F). For the sake of convenience, the P1(–102/–63) motif was designated as NRE-1, and the P1(–39/+1) motif was designated as NRE-2 in the study.

### 2.5. Repression of the MDM2 P1 Promoter by ORF50 Is Mediated through Inhibition of Sp1-Mediated Transactivation

**Figure 6:**
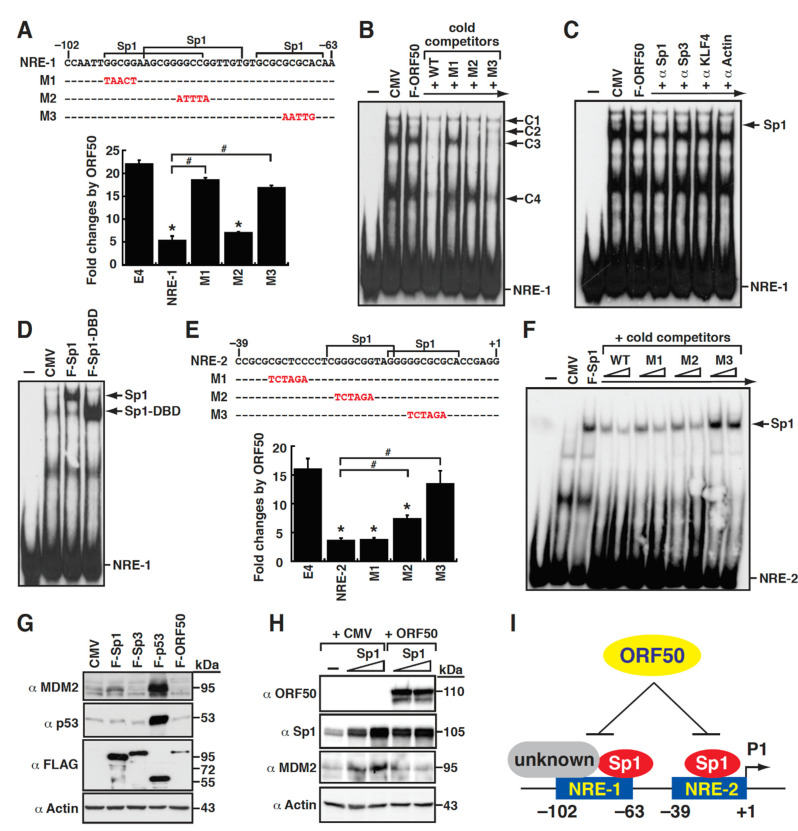
Sp1 is involved in the ORF50-mediated transcriptional repression of the P1 promoter of the MDM2 gene. (**A**) Point mutational analysis of the P1(–103/–63) promoter element (or referred to as NRE-1). The diagram shows the nucleotide sequence of NRE-1 and its mutated elements (top panel). Notably, there are three Sp1-binding sites predicted in the NRE-1 element. The responsiveness of the reporter constructs containing the NRE-1 element or its mutants (M1, M2, and M3) to ORF50 was assayed in 293T cells (bottom panel). *, *p* < 0.05, compared to pE4-luc; #, *p* < 0.05, compared to the NRE-1 group (n = 3). (**B**) EMSA analysis of the NRE-1 element using total protein extracts of 293T cells transfected with the empty vector or the ORF50 expression plasmid. Four major protein/DNA complexes (C1, C2, C3, and C4) were detected in the EMSA. Cold competitors shown in panel (**A**) were added at a concentration of a 50-fold molar excess relative to the labeled probe. (**C**) Antibody supershifting or blocking test in EMSA. Specific antibodies to Sp1, Sp3, KLF4, or actin were used for EMSA supershift or blockade assay. (**D**) Direct binding of Sp1 or the Sp1 DNA-binding domain (Sp1-DBD) to the NRE-1 element. EMSA was performed using total protein extracts of 293T cells transfected with the empty vector or the plasmid expressing the full-length Sp1 or Sp1-DBD. (**E**) Point mutational analysis of the P1(–39/+1) promoter element (or referred to as NRE-2). The nucleotide sequences of NRE-2 and its mutated elements are shown in the diagram (top panel). Note that there are two Sp1-binding sites predicted in the NRE-2 element. The responsiveness of the indicated reporter constructs to ORF50 were determined in 293T cells (bottom panel). *, *p* < 0.05, compared to pE4-luc; #, *p* < 0.05, compared to the NRE-2 group. (**F**) Direct binding of Sp1 to the NRE-2 element. EMSA was performed using total protein extracts of 293T cells transfected with the empty vector or the Sp1 expression plasmid. Cold competitors shown in panel (**E**) were added at a concentration of a 30-or 60-fold molar excess relative to the labeled probe. (**G**) Effects of Sp1, Sp3, p53, or ORF50 on the expression of MDM2 in 293T cells. 293T cells were transfected with the indicated expression plasmids, and the protein expression of MDM2 in these transfected cells was analyzed by immunoblotting. (**H**) Effect of ORF50 on the Sp1-mediated MDM2 upregulation in 293T cells. Increasing amounts of the Sp1 expression plasmid were cotransfected with the empty vector or the ORF50 expression plasmid into 293T cells for 30 h. The expression levels of MDM2, Sp1, and ORF50 in these transfected cells were determined by Western blot analysis. (**I**) Proposed model for the repressive effect of ORF50 on Sp1-mediated activation of the MDM2 P1 promoter.

As shown in Figure 5, two small elements including P1(–102/–63) and P1(–39/+1), or referred to as NRE-1 and NRE-2, in the MDM2 P1 promoter were critical for mediating ORF50 repression. We next determined whether specific cellular proteins that bind to these DNA elements were involved in ORF50-mediated repression. Sequence analysis showed that NRE-1 is a GC-rich element and contains three putative Sp1-binding sites (Figure 6A). To evaluate the importance of these predicted Sp1-binding sites for conferring ORF50 responsiveness, specific point mutations were introduced into the NRE-1/E4 reporter construct (Figure 6A). The results from luciferase reporter assays show that point mutations at the second Sp1-binding site (M2) in the NRE-1 element did not affect ORF50-mediated repression; however, mutations at the first and third Sp1-binding sites (M1 and M3) greatly impaired ORF50-mediated repression (Figure 6A). These results indicate that both the M1 and M3 sites in the NRE-1 element are critical for ORF50-mediated repression, and cellular transcription factors that bind to these two sites may potentially involve this transcriptional regulation. To identify proteins that bind to the NRE-1 element, we performed electrophoretic mobility shift assays (EMSAs) using total protein extracts of 293T cells that were transfected with the empty vector or ORF50-expressing plasmid (Figure 6B). Compared to the protein/DNA complexes formed by the control cell lysate in EMSAs, we were unable to find differences in the protein/DNA complexes formed by the ORF50-transfected cell lysates (Figure 6B). Our results imply that (1) ORF50 may not directly bind to the NRE-1 element and (2) changes in the DNA-specific binding factors induced by ORF50 may be relatively minor (such as post-translational modifications) or could not be easily detected in EMSA experiments (such as multisubunit complexes). There were four major protein/DNA complexes, designated C1 to C4, detected in EMSAs using these protein extracts (Figure 6B). When tested in competitive EMSAs using different cold competitors, we found that the C3 protein complex specifically bound to the M1 site, whereas the C2 protein complex bound to the M3 site (Figure 6B). Since Sp family proteins could bind to GC-rich sequences with similar affinity, we performed a supershift/blocking assay to determine whether specific Sp family proteins, including Sp1, Sp3, or KLF4, bound to the NRE-1 element. The addition of antibodies in EMSA experiments revealed that the antibody specific for Sp1, but not Sp3 or KLF4, substantially removed the C2 complex and partially reduced the formation of C3 complex (Figure 6C), suggesting that both C2 and C3 complexes could be Sp1-related complexes. To further verify the binding of Sp1 protein to the NRE-1 element, EMSA experiments were performed using total protein extracts of 293T cells that were transfected with the expression plasmid encoding a full-length Sp1 or the Sp1 DNA-binding domain (DBD; aa 300 to 785) (Figure 6D). Our results clearly show that Sp1 could specifically bind to the NRE-1 element, which corresponds to the C2 complex (Figure 6D).

On the other hand, a sequence analysis of the P1(–39/+1) element, also known as NRE-2, also revealed that it contains two Sp1-binding sites (Figure 6E). Specific point mutations (including M1, M2, and M3) were then introduced in the NRE-2 element. A transient reporter analysis showed that point mutations at the M1 site of the NRE-2 element did not affect its response to ORF50 (Figure 6E). However, point mutations at the Sp1-binding sites, particularly at the M3 site, in the NRE-2 element remarkably reduced their responses to ORF50 (Figure 6E, M2, and M3). In EMSA experiments, we demonstrated that Sp1 specifically bound to the NRE-2 element, and the binding of Sp1 to the NRE-2 element was mainly mediated through the M3 site because the Sp1/DNA complex could not be completed by the cold competitor with mutations at the M3 site (Figure 6F).

To determine the involvement of Sp1 in the ORF50-mediated MDM2 repression, we first clarified the function of Sp1 in MDM2 expression. As shown in Figure 6G, overexpression of F-Sp1, but not F-Sp3, could substantially increase the expression level of MDM2 in 293T cells. Since p53 is well characterized as a transcriptional activator for MDM2 expression, the transfection of 293T cells with the F-p53 expression plasmid was used as a positive control in the experiment (Figure 6G). Intriguingly, when the ORF50 expression plasmid was cotransfected with the Sp1 expression plasmid in 293T cells, the promoting effect of Sp1 on MDM2 expression was significantly blocked by ORF50 (Figure 6H). Overall, our findings suggest that the repression of the MDM2 P1 promoter by ORF50 could be mediated, at least in part, through inhibiting Sp1-dependent transactivation (Figure 6I).

### 2.6. Defining the ORF50-Dependent Negative Response Element in the MDM2 P2 Promoter

**Figure 7:**
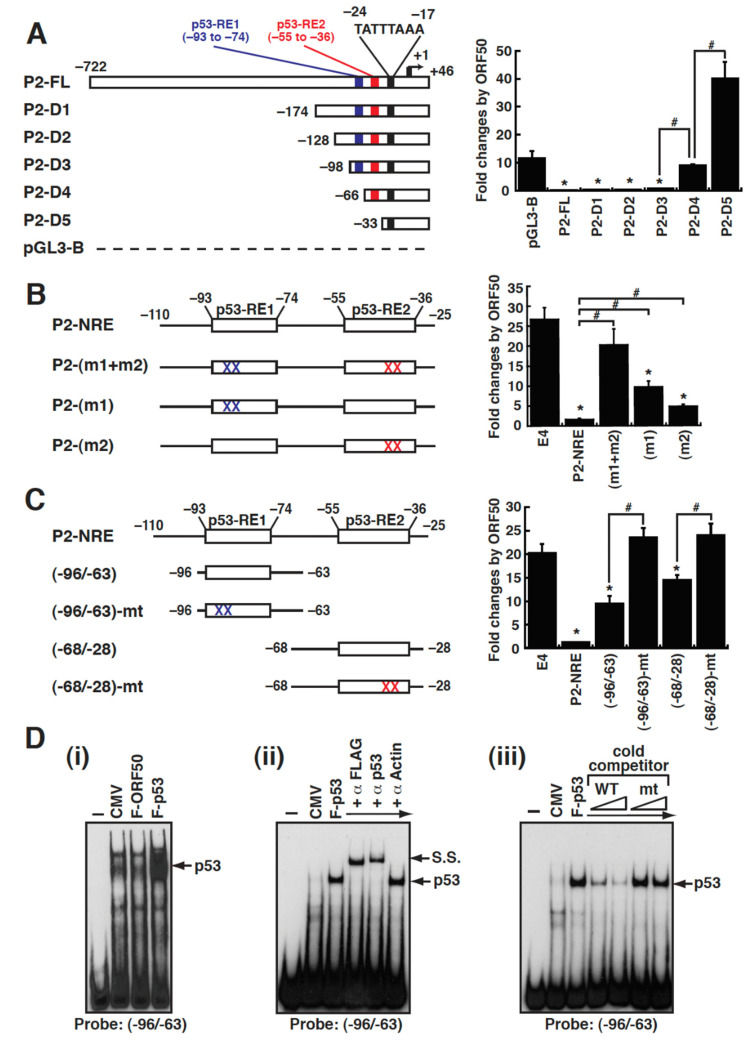
The p53-responsive elements in the P2 promoter of the MDM2 gene are critically required for the ORF50-mediated repression. (**A**) Deletion analysis of the MDM2 P2 promoter. The reporter constructs with the P2 promoter region from –722 to +46 (P2-FL) and its 5′ deletions are shown in the diagram (left panel). Notably, the P2 promoter contains a TATA-like box located between –24 to –17 and two p53-responsive elements (p53-RE1 and p53-RE2) located from –93 to –74 and from –55 to –36, respectively. Thirty hours after cotransfection, the fold change of the indicated reporter constructs in responding to ORF50 in 293T cells was determined by luciferase reporter assays (right panel). *, *p* < 0.05, compared to pGL3-Basic; #, *p* < 0.05, compared to the indicated reporter constructs (n = 3). (**B**) Point mutational analysis of the P2(–110/–25) promoter element. The left diagram shows the P2(–110/–25) promoter element (or referred to as P2-NRE) and its mutated elements that contain point mutations at the p53-RE1 or p53-RE2 motif or both motifs. These P2 promoter elements in pE4-luc were tested for their responses to ORF50 in 293T cells (right panel). *, *p* < 0.05, compared to pE4-luc; #, *p* < 0.05 compared to P2-NRE. (**C**) Responsiveness of the reporter constructs harboring the p53-RE1 or p53-RE2 motif to ORF50. The left diagram shows the wild-type or mutant p53-RE motifs cloned into pE4-luc. The luciferase reporter assay was performed in 293T cells by cotransfection of the indicated reporter constructs with the empty vector or the ORF50-expressing plasmid. Data are indicated as fold change of the luciferase activity of the reporter construct in the presence of ORF50 over the luciferase activity in the absence of ORF50 (right panel). *, *p* < 0.05, compared to pE4-luc; #, *p* < 0.05, compared to the indicated reporter constructs. (**D**) EMSAs of the P2(–96/–63) element using total protein extracts of 293T cells transfected with the expressing plasmid encoding FLAG-tagged p53 (F-p53). The binding of F-p53 to the P2(–96/–63) motif of the P2 promoter was demonstrated by EMSAs (i–iii) in combination with antibody supershift assay (ii) and competition assay (iii). The F-p53-specific complex and the supershifted (S.S.) complex are indicated. The cold competitors used in (iii) were the P2(–96/–63) and P2(–96/–63)-mt elements as shown in (**C**).

The P2 promoter of the MDM2 gene is known as a transcriptional target of p53 [20,22], which contains a TATA box (TATTTAAA) located from –24 to –17 and two tandem p53-responsive elements (p53-RE1 and p53-RE2) located from –93 to –74 and from –55 to –36, respectively (Figure 7A). To map the ORF50-dependent negative response element in the P2 promoter, a series of 5′ deletions of the P2 promoter were cloned into pGL3-Basic. A transient reporter analysis revealed that two regions in the P2 promoter, located from –98 to –66 and from –66 to –33, were critically required for ORF50-mediated repression (Figure 7A). Interestingly, the identified ORF50-dependent negative response regions were coincident with the location of the two known p53-responive elements (Figure 7A).

To verify the identified ORF50-dependent negative response regions in the P2 promoter, the promoter fragment from –110 to –25, which covers both p53-REs, were constructed into pE4-luc. Compared to pE4-luc, the resultant reporter construct P2(–110/–25)/E4 was significantly repressed by ORF50 in 293T cells (Figure 7B), confirming that the P2(–110/–25) element was an ORF50-dependent negative response element. To determine whether the p53-RE1 and p53-RE2 motifs in the P2(–110/–25) region were required for conferring ORF50-mediated repression, point mutations at p53-RE1, p53-RE2, or both p53-REs were made in the reporter construct P2(–110/–25)/E4 (Figure 7B). A transient reporter assays showed that point mutations at both p53-REs in the promoter element almost completely abolished the ORF50-dependent repression (Figure 7B). However, point mutations at either p53-RE1 or p53-RE2 in the P2(–110/–25) element still retained a partial ability to confer the ORF50-dependent repression (Figure 7B), suggesting that both the p53-RE1 and p53-RE2 motifs contributed to ORF50-mediated repression. Based on the results from Figure 7B, we additionally noticed that the p53-RE1 motif seemed to have a better ability to respond to ORF50 than the p53-RE2 motif.

To further determine whether the p53-RE1 or p53-RE2 motif alone was functional for responding to ORF50, the P2(–98/–63) and P2(–68/–28) elements that cover the p53-RE1 and p53-RE2 motifs, respectively, were individually cloned into pE4-luc (Figure 7C). Additionally, the corresponding elements with point mutations at the p53-RE1 or p53-RE2 site were also cloned into pE4-luc as negative controls (Figure 7C). Compared to pE4-luc, both the P2(–98/–63)/E4 and P2(–68/–28)/E4 reporter constructs still showed a significant ORF50-dependent repression, although the abilities of these two reporter constructs were weaker than that of the P2(–110/–25)/E4 reporter construct (Figure 7C). Importantly, the p53-RE1- and p53-RE2-mutated reporter constructs completely lost their responses to ORF50 (Figure 7C), demonstrating that these two p53-RE elements were the functional ORF50-dependent negative response elements. To verify the binding of p53 to the identified ORF50-dependent negative response element, the P2(–96/–63) element was chosen as a DNA probe in EMSAs. By using the protein extract of 293T cells that were transfected with the plasmid expressing FLAG-tagged p53 (F-p53), we confirmed that F-p53 could specifically bind to the P2(–96/–63) element in EMSA experiments together with supershift and competition tests (Figure 7D(i–iii)).

### 2.7. Repression of the MDM2 P2 Promoter by ORF50 Is Mediated through Inhibiting p53 Transactivation

**Figure 8:**
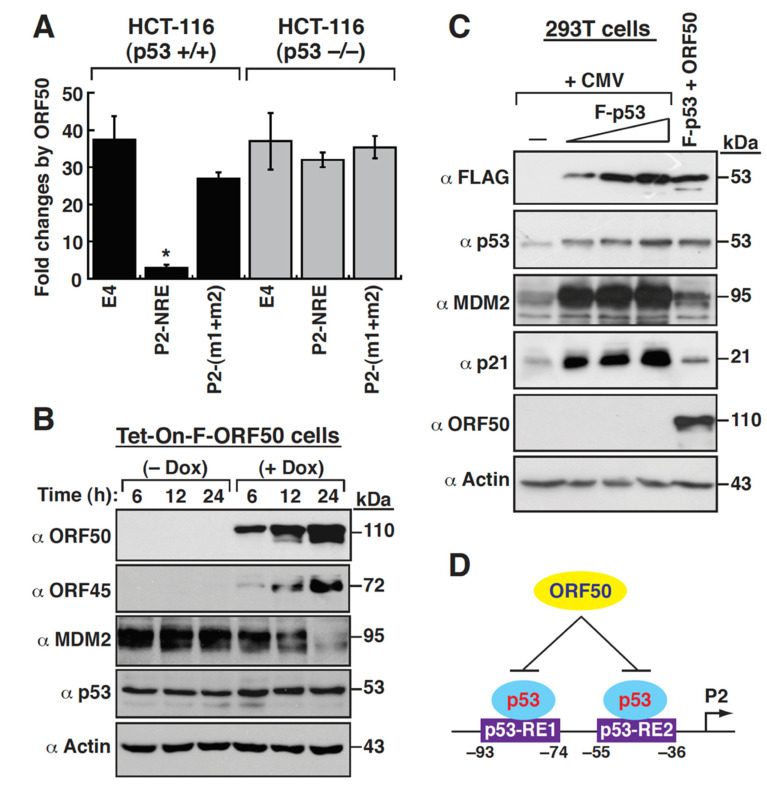
Inhibition of p53-mediated transactivation by ORF50. (**A**) Responsiveness of different reporter constructs including pE4-luc, P2-NRE/E4, and P2(m1+m2)/E4 to ORF50 in p53-wild-type and p53-null HCT116 cells. After cotransfection with the indicated reporter constructs and the ORF50-expressing vector or the empty vector control in HCT116 (p53+/+) cells or in HCT116 (p53−/−) cells for 30 h, cells were harvested for luciferase reporter assays. Fold change was calculated as the luciferase activity of the indicated reporter construct in the presence of the ORF50 expression plasmid divided by that in the presence of the empty vector control (CMV). *, *p* < 0.05, for results compared to those with pE4-luc. (**B**) Western blot analysis of the expression levels of MDM2 and p53 in HH-B2(Tet-On-F-ORF50) cells after ORF50 overexpression. HH-B2(Tet-On-F-ORF50) cells that were left untreated or treated with doxycycline for 6, 12, and 24 h were examined for the expression of MDM2, p53, ORF50, and ORF45 by immunoblotting. (**C**) Effect of ORF50 on p53-mediated MDM2 upregulation in 293T cells. Cells were cotransfected with the plasmid expressing FLAG-tagged p53 (F-p53) and the empty control vector or the ORF50 expression plasmid for 30 h, and the expression levels of p53, MDM2, p21, and ORF50 in these transfected cells were examined by Western blot analysis. (**D**) Proposed model for the repression of p53-mediated MDM2 upregulation by ORF50.

Based on the above results, ORF50 might repress the MDM2 P2 promoter activity through repressing the function of p53. To elucidate the role of p53 protein involved in the ORF50-mediated MDM2 repression, the p53-wild-type (p53^+/+^) and p53-null (p53^−/−^) HCT116 cells were used to analyze the MDM2 P2 promoter activity in response to ORF50. When the P2(−110/−25)/E4 reporter constructs with or without p53-RE mutations were cotransfected with the ORF50 expression plasmid or the empty vector into HCT116 (p53^+/+^) cells, only the wild-type P2(−110/−25)/E4 reporter construct, but not the p53-RE-mutated counterpart, showed an ORF50-mediated repression (Figure 8A). However, in HCT116 (p53^−/−^) cells, neither the wild-type P2(−110/−25)/E4 reporter construct nor the p53-RE-mutated reporter construct was repressed by ORF50 (Figure 8A). These results strongly suggest that p53 plays a key role in ORF50-dependent repression of the MDM2 P2 promoter. Although p53 was critically involved in the ORF50-mediated MDM2 downregulation, ORF50 did not appear to affect the overall protein levels of p53 in cells. As shown in Figure 2B and Figure 6G, overexpression of F-ORF50 in 293T cells did not influence the expression level of p53. Additionally, the induction of ORF50 expression in HH-B2(Tet-On-F-ORF50) cells by doxycycline, which led to MDM2 downregulation, also did not significantly alter p53 expression levels (Figure 8B). To further confirm the involvement of p53 in ORF50-mediated MDM2 downregulation, the ORF50 expression plasmid was cotransfected with the F-p53 expression plasmid in 293T cells. Western blot analysis revealed that increasing amounts of F-p53 expression in 293T cells could markedly increase the expression levels of MDM2 and p21, another transcriptional target of p53 [32] (Figure 8C). However, the promoting effect of F-p53 on the MDM2 and p21 expression could be remarkably repressed by ORF50 (Figure 8C). Based on these results, we proposed that ORF50 might repress the MDM2 P2 promoter via inhibiting p53-mediated transactivation (Figure 8D).

## 3. Discussion

ORF50 is a multifunctional protein critically involved in the latent-to-lytic switch of KSHV [5,33]. In this report, we show that ORF50 protein can downregulate the expression of MDM2, a negative regulator of KSHV lytic reactivation, through repressing both the P1 and P2 promoters of the MDM2 gene. Furthermore, we reveal that ORF50 protein possesses antagonistic actions on the transactivation functions of Sp1 and p53. These findings emphasize the complexity of numerous distinct functions of ORF50 in the viral lytic cycle and may help to provide new insights into the regulation of cellular gene expression that supports optimal viral replication.

### 3.1. Negative Reciprocal Regulation between ORF50 and MDM2

Since latency is the default outcome of KSHV infection, this virus may have evolved many different strategies to prevent the expression of ORF50 and the occurrence of the viral lytic replication. For blocking the ORF50 expression, in addition to the transcriptional repression of the ORF50 gene promoter by modulating specific transcriptional factors [5], we previously reported that MDM2 could lower the basal protein level of ORF50 by promoting its protein degradation [25]. Therefore, MDM2 acts as a negative regulator of ORF50 expression. In the present study, we conversely showed that abundant ORF50 expression could lead to MDM2 downregulation in PEL cells and in 293T cells (Figure 1 and Figure 2). These findings strongly imply that MDM2 and ORF50 can create a reciprocal negative regulation loop in which MDM2 decreases the ORF50 protein level, whereas abundant ORF50 expression inhibits the MDM2 promoter activity. The balance between MDM2 and ORF50 expression may be a key determinant for the latent-to-lytic switch of KSHV. At the early stages of lytic replication, we think that MDM2 downregulation could be attributed to an abundant expression of ORF50. However, we noticed that sustained downregulation of MDM2 could be detected at the late lytic stages even in the absence of ORF50 protein (Figure 1). It may be possible that other viral lytic proteins can separately coordinate MDM2 downregulation during the late lytic stages. For future research, it would be interesting to determine whether and how MDM2 is downregulated by other viral lytic proteins.

### 3.2. ORF50 Protein Represses the P1 and P2 Promoters of the MDM2 Gene

The expression levels of MDM2 are highly regulated at both the transcriptional and post-transcriptional levels in cells under normal and pathological conditions [18,20]. Herein, we demonstrate that ORF50 represses MDM2 expression mainly at the transcriptional level, but not at the post-transcriptional level (Figure 2). There are two promoters, P1 and P2, that control the transcription of the MDM2 gene. We found that both the P1 and P2 promoters are repressed by ORF50 (Figure 3 and Figure 4). Since ORF50 is considered as a transcriptional activator, the repressive effect of ORF50 on the P1 and P2 promoters of the MDM2 gene was unexpected. Additionally, when the ORF50 deletion mutants were used in repression assays, we surprisingly found that the F-ORF50(357-691) construct, which lacks the ORF50 DNA-binding domain, still retained full ability to repress the P2 promoter, but not the P1 promoter (Figure 4). These findings lead to two important conclusions. First, the regulatory mechanisms by which ORF50 represses the P1 and P2 promoters of the MDM2 gene may be different. Second, the repression of the MDM2 P2 promoter by ORF50 may be independent of ORF50′s transactivation function.

For elucidating the potential mechanism of repression of the MDM2 P1 promoter by ORF50, we mapped two ORF50-dependent negative response elements located from –102 to –63 (designated as NRE-1) and from –39 to +1 (designated as NRE-2), respectively (Figure 5). To our knowledge, this is the first study to point out the potential importance of the NRE-1 and NRE-2 elements in the MDM2 P1 promoter activity. These two ORF50-dependent negative response elements could be bound by Sp1 and several unknown proteins (Figure 6). Among these binding proteins, Sp1 may be the only protein specifically bound to both the NRE-1 and NRE-2 elements. Importantly, we demonstrated that the transfection of Sp1 into 293T cells could greatly enhance the expression of MDM2, indicating that Sp1 is indeed a positive regulator of MDM2 expression (Figure 6G). However, the promoting effect of Sp1 on the upregulation of MDM2 could be dramatically inhibited by ORF50 (Figure 6H). Due to the fact that ORF50 expression in 293T cells or in PEL cells could not significantly change the expression levels of Sp1 (Supplementary Figure S1), our findings therefore suggest that ORF50 may repress the MDM2 P1 promoter through the inhibition of the Sp1-mediated transactivation.

For the repression of the MDM2 P2 promoter by ORF50, we found that the ORF50-depedent negative response element overlaps with two known p53-REs in the P2 promoter (Figure 7). Mutation or deletion analysis demonstrated that each p53-RE (p53-RE1 or p53-RE2) was functional to confer the ORF50-mediated repression. (Figure 7B,C). Furthermore, the ORF50-mediated repression of the P2 promoter was detected only in p53-wild-type HCT116 cells, but not in p53-null HCT116 cells (Figure 8A), indicating that p53 plays an important role in the ORF50-mediated MDM2 repression. Particularly, we found that overexpression of p53 in 293T cells could activate the expression levels of both MDM2 and p21; however, the p53-mediated activation of MDM2 and p21 could be markedly repressed by ORF50 (Figure 8C). Since ORF50 expression did not affect the expression level of p53 in 293T cells (Figure 2B and Figure 6G) and in PEL cells (Figure 8B), we conclude that ORF50 suppresses the P2 promoter of the MDM2 gene through repressing the p53-mediated transactivation.

Although the detailed mechanisms by which ORF50 represses the P1 and P2 promoters of the MDM2 gene are not fully elucidated, we currently do not think that ORF50 could directly bind to the MDM2 P1 and P2 promoters according to our EMSA experiments (Figure 5B,C, Figure 7D and Figure S2). Several possibilities may be proposed to explain how ORF50 inhibits Sp1- or p53-mediated transactivation indirectly. Firstly, ORF50 may substantially weaken the transactivation activity or the DNA-binding activity of Sp1 or p53 in cells by modulating their post-translational modifications (such as phosphorylation, glycosylation, acetylation, or sumoylation). Secondly, ORF50 may compete for Sp1 or p53 binding to the limiting coactivators (such as p300 and CBP) and therefore reduce the availability of coactivators for Sp1 or p53. Thirdly, ORF50 may activate the expression of various corepressors (such as HDAC1) or other negative regulators that physically associate with Sp1 or p53. In future research, elucidation of these possible mechanisms will be important to provide further insights into the molecular complexity of the ORF50-mediated gene expression.

## 4. Materials and Methods

### 4.1. Cell Cultures, Reagents, and Transfections

HH-B2 [34] and BC3 [35] are KSHV-infected primary effusion lymphoma (PEL) cell lines. Both PEL cell lines were cultured in RPM1 1640 medium (#11875085; Gibco, Thermo Fisher Scientific, Waltham, MA, USA) supplemented with 15% fetal bovine serum (FBS; #10437028; Gibco, Thermo Fisher Scientific, Waltham, MA, USA). 293T cells, a human embryonic kidney cell line [36], were cultured in high-glucose Dulbecco’s modified Eagle medium (DMEM; #11965084; Gibco, Thermo Fisher Scientific, Waltham, MA, USA) supplemented with 10% FBS. 293T(BAC16) cells that harbor KSHV BAC16 DNA [37] were cultured in high-glucose DMEM supplemented with 10% FBS and 1200 μg/mL hygromycin (#400052; Merck Millipore, Burlington, MA, USA). HH-B2(Tet-On-F-ORF50) is a stable HH-B2 cell clone that contains a doxycycline-regulated ORF50 gene [38]. HH-B2(Tet-On-F-ORF50) cells were grown in RPMI 1640 medium containing 15% FBS, 400 μg/mL hygromycin, and 100 μg/mL G418 (#1720; Sigma-Aldrich, St. Louis, MO, USA). For induction of the exogenous ORF50 gene in HH-B2(Tet-On-F-ORF50) cells, doxycycline (#D1822; Sigma-Aldrich, St. Louis, MO, USA) was added at 1 μg per ml. For viral lytic induction, both HH-B2 and BC3 cells were treated with 3 mM sodium butyrate (#B5887; Sigma-Aldrich, St. Louis, MO, USA). Human colon carcinoma cell lines, HCT116 (p53^+/+^) and HCT116 (p53^−/−^), were kindly provided by Chung-Sheng Shi (Chang Gung University, Taiwan). These two HCT116 cell lines were cultured in RPMI 1640 medium containing 10% FBS. All transient transfection experiments were carried out using Lipofectamine 2000 or Lipofectamine 3000 transfection reagent (#11668019 or #L3000008; Thermo Fisher Scientific, Waltham, MA, USA) according to the manufacturer’s recommendations.

### 4.2. Plasmid Construction

The expression plasmids including pCMV-FLAG-ORF50, pCMV-FLAG-ORF50(1-564), pCMV-ORF50(357-691), pCMV-FLAG-Sp1, pCMV-FLAG-Sp1(300-785), and pCMV-FLAG-Sp3, as well as the reporter construct pE4-luc were described previously [14,39,40]. To generate the P1-Luc and P2-Luc reporter constructs, the corresponding MDM2 promoter regions were first amplified by PCR using total genomic DNA from BJAB cells as a template and then cloned into pGL3-Basic (#E1751; Promega, Madison, WI, USA). For the generation of deletion mutants in the P1-Luc and P2-Luc reporter constructs, truncated promoter fragments were inserted into pGL3-Basic. Additionally, several small promoter elements, which were produced either by PCR amplification or by the annealing of two complementary oligonucleotides, were cloned into E4-luc, a luciferase reporter construct with an adenovirus E4 minimal promoter. The expression plasmid pCMV-FLAG-p53 that encodes FLAG-tagged p53 (GenBank ID: NM_000546) was obtained from GenScript Bioteck (#OHu20059D; Piscataway, NJ, USA), whereas the expression plasmid pCMV-HA-KLF4 that encodes HA (hemagglutinin)-tagged KLF4 (GenBank ID: AF105036) was purchased from Addgene (#34593; Watertown, MA, USA).

### 4.3. Western Blot Analysis

Detailed protocol for Western blot analysis of protein samples was described previously [41]. The anti-ORF50 polyclonal antibody used in the study was generated in our laboratory using purified ORF50(333–691) as an immunogen. Antibodies to MDM2 (M4308; Sigma-Aldrich, St. Louis, MO, USA), K8 (sc-57889; Santa Cruz, Dallas, TX, USA), ORF45 (sc-53883; Santa Cruz, Dallas, TX, USA), K8.1 (sc-65446; Santa Cruz, Dallas, TX, USA), FLAG (A8592; Sigma-Aldrich, St. Louis, MO, USA), p53 (sc-126; Santa Cruz, Dallas, TX, USA), Sp1 (CS200631; Millipore, Burlington, MA, USA), p21 (sc-6246; Santa Cruz, Dallas, TX, USA), and actin (sc-47778; Santa Cruz, Dallas, TX, USA) were purchased commercially.

### 4.4. Quantitative Reverse Transcription PCR (RT-qPCR)

Protocols for total RNA extraction from cells, reverse transcription, and quantitative PCR analysis were mentioned previously [38]. In brief, total RNAs were isolated from cells using RNeasy kits (#74104; QIAGEN, Germantown, MD, USA) in combination with the RNase-free DNase set (#79254; QIAGEN, Germantown, MD, USA). First-strand cDNA was synthesized from total RNAs using RevertAid First Strand cDNA Synthesis kit (#K1622; Thermo Fisher Scientific, Waltham, MA, USA). Quantitative PCR was performed using iQ SYBR Green Supermix (#170882; Bio-Rad, Hercules, CA, USA) according to the manufacturer’s recommendations. The PCR primers used in the study were following: 5′-TCACCTTGAAGGTGGGAGTGATCA-3′ (positioned in exon 6) and 5′-CTTCTGTCTCACTAATTGCTCTCC-3′ (positioned in exon 7) for total MDM2 mRNA; 5′-GGAAACTGGGGAGTCTTGAGGGAC-3′ (positioned in exon 1a) and 5′-TCCGAAGCTGGAATCTGTGAGGTG-3′ (positioned in exon 2) for P1-derived mRNA; 5′-GATTGGAGGGTAGACCTGTGGGCA-3′ (positioned in exon 1b) and 5′-TCCGAAGCTGGAATCTGTGAGGTG-3′ (positioned in exon 2) for P2-derived mRNA; 5′-GGATGTAAAGGATGGAAAATACA-3′ and 5′-TCCAGGTCTTCACGGAGCTTGTT-3′ for 18S rRNA. It is worth noting that the MDM2 gene consists of 12 exons (GenBank: AF527840.1), including exon 1a, exon 1b, and exons 2–11. Relative changes of MDM2 mRNA transcripts were quantified by normalizing to 18S rRNA.

### 4.5. Cycloheximide Chase Assay

HH-B2(Tet-On-F-ORF50) cells were first cultured in medium with or without doxycycline (1μg/mL) for 16 h and then treated with 75 μg/mL cycloheximide (#C7688; Sigma-Aldrich, St. Louis, MO, USA) to prevent de novo protein synthesis. After 0.5, 1, 2, and 3 h of treatment with cycloheximide, cells were prepared and subjected to Western blot analysis. Relative levels of MDM2 normalized to actin were quantified in the study.

### 4.6. Luciferase Reporter Assays

293T cells (1.5 × 10^5^) and HCT-116 cells (3 × 10^5^) were initially seeded on 24-well plates for 18–24 h and then were cotransfected with a constant amount of the luciferase reporter plasmid (300 ng) and the effector plasmid (300 ng). Cells were typically harvested 24–30 h after transfection, and the reporter assays were performed using the luciferase reporter assay system (#E1501; Promega, Madison, WI, USA) according to the manufacturer’s instructions. Fold change was calculated as the luciferase activity of the reporter construct in the presence of effectors divided by that in the absence of effectors. All data presented in the study were obtained from at least three independent experiments.

### 4.7. Electrophoretic Mobility Shift Assay (EMSA)

Detailed protocols for EMSAs were described previously [14,42]. Briefly, for preparation of total protein extracts, cells were lysed in a buffer containing 0.42 M NaCl, 20 mM HEPES (pH 7.5), 25% glycerol, 1.5 mM MgCl_2_, 0.2 mM EDTA, 1 mM dithiothreitol, 1 mM phenylmethylsulfonyl fluoride, and 2 μg aprotinin per ml. All DNA probes were end-labeled with biotin-11-UTP using terminal deoxynucleotidyl transferase (#89818; Pierce, Thermo Fisher Scientific, Waltham, MA, USA). In the EMSA binding reaction, 10–15 μg of total protein extract was mixed with 1 ng DNA probe in a solution containing 10 mM HEPES (pH 7.5), 50 mM NaCl, 2 mM MgCl2, 2.5 μM ZnSO_4_, 0.5 mM EDTA, 1 mM dithiothreitol, 15% glycerol, and 0.3 μg poly(dI-dC) in a total volume of 20 μL. For competition assays, unlabeled cold competitors were added to the initial reaction mix. Antibodies to Sp1 (CS200631; Millipore, Burlington, MA, USA), Sp3 (sc-365220; Santa Cruz, Dallas, TX, USA), KLF4 (sc-393462; Santa Cruz, Dallas, TX, USA), p53 (sc-126; Santa Cruz, Dallas, TX, USA), FLAG (SI-A2220; Sigma-Aldrich, St. Louis, MO, USA), and actin (sc-47778; Santa Cruz, Dallas, TX, USA) were used in supershift/blocking assays

### 4.8. Statistical Analysis

All data presented in the study are expressed as mean ± standard error of mean (SEM). Statistical analysis was performed using SPSS (Statistical Package for the Social Sciences, IBM, Armonk, NY, USA) software version 18.0. Significant differences between groups were evaluated by Student’s *t* test, and *p* < 0.05 was considered statistically significant.

## 5. Conclusions

In summary, we propose that there is a reciprocal negative regulation loop between ORF50 and MDM2 and find that ORF50 can repress MDM2 expression through the inhibition of Sp1- and p53-mediated transactivation. Further research may be needed to elucidate the detailed mechanisms by which ORF50 negatively regulates the transcriptional functions of Sp1 and p53.

## Data Availability

All data presented in the study are available upon request from the corresponding author.

## Supplementary Materials

The supporting information can be downloaded at: https://www.mdpi.com/article/10.3390/ijms23158673/s1.

## Abbreviations

KSHV	Kaposi’s sarcoma-associated herpesvirus
HHV8	human herpesvirus 8
ORF50	open reading frame 50
MDM2	murine double-minute 2 homolog
KS	Kaposi’s sarcoma
PEL	primary effusion lymphoma
RTA	replication and transcription activator
siRNA	small interfering RNA
SB	sodium butyrate
RT-qPCR	quantitative reverse transcription polymerase chain reaction
NRR	negative response region
NRE	negative response element
EMSA	electrophoretic mobility shift assay
DBD	DNA-binding domain
NLS	nuclear localization signal
