# Supplementary material for: Kaposi’s Sarcoma-Associated Herpesvirus ORF50 Protein Represses Cellular MDM2 Expression via Suppressing the Sp1- and p53-Mediated Transactivation
Source: Int J Mol Sci. 2022 Aug 4;23(15):8673. doi: 10.3390/ijms23158673 (PMC9369062; doi:10.3390/ijms23158673)
Supplement: Supplementary file 1 [file ijms-23-08673-s001.zip › ijms-1792399-supplementary.pdf]

SUPPLEMENTAL INFORMATION

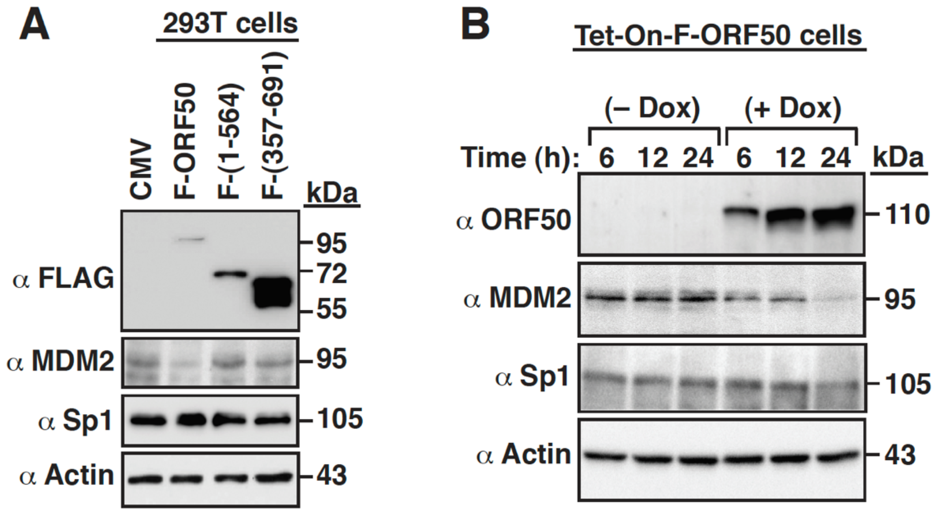

Supplementary Figure S1. Overexpression of ORF50 does not substantially affect the expression of Sp1 in 293T cells (A) and in HH-B2(Tet-On-F-ORF50) cells (B).

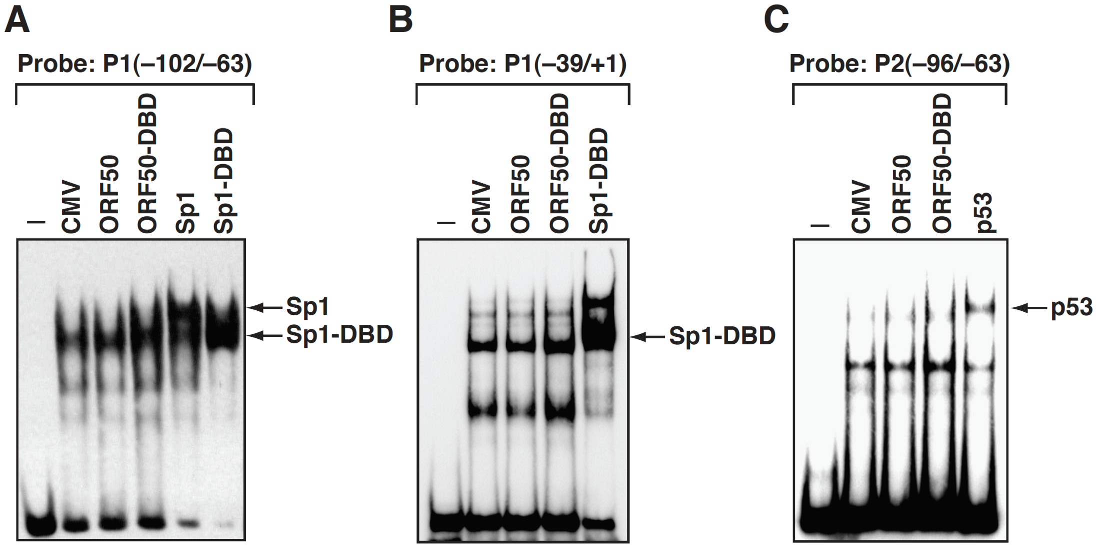

Supplementary Figure S2. Both the full-length ORF50 protein and the DNA-binding domain (DBD; aa 1 to 490) of ORF50 do not exhibit an ability to bind the ORF50-dependent negative response elements in the P1 and P2 promoters of the MDM2 gene. EMSA experiments were performed using the P1(–102/–63), P1(–39/+1) or P2(–96/–63) element as probes (A–C), and the protein extracts of 293T cells transfected with the expression plasmids encoding the full-length ORF50, ORF50-DBD, the full-length Sp1, Sp1-DBD or p53.
